# Hemoglobin Is a Co-Factor of Human Trypanosome Lytic Factor

**DOI:** 10.1371/journal.ppat.0030129

**Published:** 2007-09-07

**Authors:** Justin Widener, Marianne Jensby Nielsen, April Shiflett, Søren Kragh Moestrup, Stephen Hajduk

**Affiliations:** 1 Program in Pathobiology, Brown University, Providence, Rhode Island, United States of America; 2 Department of Biochemistry and Molecular Biology, University of Georgia, Athens, Georgia, United States of America; 3 Department of Medical Biochemistry, University of Aarhus, Aarhus, Denmark; Washington University School of Medicine, United States of America

## Abstract

Trypanosome lytic factor (TLF) is a high-density lipoprotein (HDL) subclass providing innate protection to humans against infection by the protozoan parasite *Trypanosoma brucei brucei.* Two primate-specific plasma proteins, haptoglobin-related protein (Hpr) and apolipoprotein L-1 (ApoL-1), have been proposed to kill *T. b. brucei* both singularly or when co-assembled into the same HDL. To better understand the mechanism of *T. b. brucei* killing by TLF, the protein composition of TLF was investigated using a gentle immunoaffinity purification technique that avoids the loss of weakly associated proteins. HDL particles recovered by immunoaffinity absorption, with either anti-Hpr or anti-ApoL-1, were identical in protein composition and specific activity for *T. b. brucei* killing. Here, we show that TLF-bound Hpr strongly binds Hb and that addition of Hb stimulates TLF killing of *T. b. brucei* by increasing the affinity of TLF for its receptor, and by inducing Fenton chemistry within the trypanosome lysosome*.* These findings suggest that TLF in uninfected humans may be inactive against *T. b. brucei* prior to initiation of infection. We propose that infection of humans by *T. b. brucei* causes hemolysis that triggers the activation of TLF by the formation of Hpr–Hb complexes, leading to enhanced binding, trypanolytic activity, and clearance of parasites.

## Author Summary

African trypanosomes are parasites that can infect a wide range of mammals, including domestic animals and humans. Several hundred thousand humans are infected with African sleeping sickness, but this number would be much higher if not for a natural defense molecule found in human blood. The trypanosome lytic factor (TLF) is a minor subclass of high-density lipoprotein that contains two proteins found only in primates, apolipoprotein L-1 and haptoglobin-related protein (Hpr). In this paper, we show that Hpr contributes to TLF toxicity to trypanosomes because it binds hemoglobin (Hb). We found that when Hb is bound to TLF, it is rapidly taken up by the parasite and activated within the acidic environment of the parasite's digestive organelle, the lysosome. Within the lysosome, Hb releases iron, inducing a chemical reaction that produces free radicals that damage membranes and contributes to trypanosome killing. Usually, free Hb is rapidly cleared from the circulation of mammals because of the organ damage free Hb can cause. Trypanosome infection results in breakage of red blood cells and the release of large amounts of Hb. We postulate that trypanosome infection causes increased vascular levels of Hb, resulting in the formation of TLF–Hb complexes that may be important in “arming” the human innate immune system to clear the circulation of certain African trypanosomes.

## Introduction

African trypanosomes are blood parasites of mammals in sub-Saharan Africa that cause chronic wasting diseases in both humans and domestic animals [1]. The three subspecies of Trypanosoma brucei are defined by their host range, geographical distribution, and course of disease [1–3]. *Trypanosoma brucei rhodesiense* and *Trypanosoma brucei gambiense* infect humans and cause African sleeping sickness, while *Trypanosoma brucei brucei* infects non-primate mammals and causes nagana in cattle. All African trypanosomes are able to evade the host adaptive immune system through a process called antigenic variation, which is a consequence of periodic changes in the variant surface glycoprotein that covers the entire parasite [4]*. T. b. brucei* does not cause human disease because of its susceptibility to an innate immune activity in human serum. This protection is conferred by trypanosome lytic factor (TLF), a minor subclass of human high-density lipoprotein (HDL) [5–7].

TLF contains apolipoprotein A-I (ApoA-1), a protein found in all subclasses of HDL, and two proteins, haptoglobin-related protein (Hpr) and apolipoprotein L-1 (ApoL-1) that are unique to primates [8–19]. Both Hpr and ApoL-1 have been reported to be toxic to *T. b. brucei* [8,14]. The cellular pathway for TLF killing of *T. b. brucei* initiates with binding of TLF to high-affinity receptors located in the flagellar pocket of the parasite [20,21]. Bound TLF is endocytosed via coated vesicles and traffics to the parasite lysosome. Within the acidified lysosome, TLF is activated and causes parasite lysis [22–24]. ApoL-1 and Hpr have been proposed to have different mechanisms of toxicity and may act synergistically. ApoL-1 is a colicin-like protein that kills trypanosomes through the formation of ion pores [10,25–29]. Hpr is a hemoglobin (Hb)-binding protein that has been proposed to induce an iron-dependent, Fenton-like reaction within the acidic lysosome of *T. b. brucei* that leads to the formation of free radicals and peroxidation of the lysosomal membranes [15,24,30]. When ApoL-1 and Hpr are present in the same HDL particle, the specific activity for *T. b. brucei* killing is enhanced 800-fold [14].

Hpr is 91% identical to haptoglobin (Hp), an abundant (∼0.45–3 mg/ml in normal human serum) acute phase serum protein, possessing very high affinity for Hb [31]. Complexes of Hp and Hb that form when Hb is released from erythrocytes undergoing intravascular hemolysis are removed from the circulation by the CD163 scavenger receptor [32]. In contrast to Hp–Hb, the Hpr–Hb complex does not bind CD163 [31,33], and the Hpr serum concentration appears to be unaffected by hemolysis [32,33]. A role for Hb in trypanolysis has previously been speculated but this view was subsequently debated [12,15,30]. In light of this, the biological significance of the recently reported high-affinity binding of recombinant Hpr to Hb remains enigmatic.

In the present study we have re-investigated a potential function of Hb in relation to TLF and trypanolysis. Hb was absent from TLF purified from freshly collected human plasma using a gentle immunoabsorption protocol. Nevertheless, Hb bound to purified native Hpr, as well as TLF, with high-affinity and stimulated *T. b. brucei* killing in vitro. The association of Hb with Hpr significantly increased TLF binding to *T. b. brucei*. Furthermore, both an iron chelator and a free radical scavenger inhibited cell lysis, suggesting a direct role for Hb-derived iron in *T. b. brucei* killing by TLF. A combination of the ion pore–forming activity of ApoL-1 and free radical production of Hpr–Hb may account for the synergy observed in HDLs containing these proteins.

## Results

### Analysis of TLF Protein Components

In order to define the protein composition of native TLF, freshly prepared human plasma was fractionated by “one-step” immunoaffinity chromatography with antibodies against Hpr or ApoL-1. Unlike the “two-step” method that initially uses high-salt density ultracentrifugation followed by immunoaffinity chromatography to purify TLF, the one-step method eliminates the possible loss of apolipoproteins during the high-salt centrifugation step [34]. These methods allowed us to make two comparisons: First, a comparison of proteins purified by the one-step purification protocol with proteins purified by the two-step purification protocol. Second, it allowed us to compare the composition of TLF purified by the one-step purification method using antibodies to ApoL-1 or Hpr.

**Figure 1:**
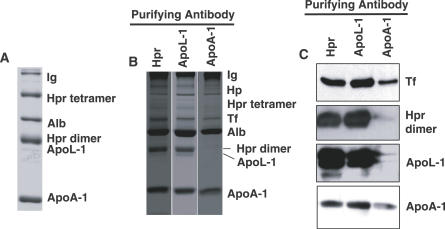
Analysis of Differentially Purified Human Serum HDLs (A) TLF analyzed by non-reducing 10% SDS-PAGE and Coomassie brilliant blue staining after purification by a two-step purification process that includes high-salt density centrifugation and antibody affinity chromatography with anti-Hpr. The presence of albumin (Alb) and immunoglobulin (Ig) are indicated. (B) HDL purified from serum using a single-step affinity purification method with antibodies to ApoL-1, Hpr, or ApoA-1 and analyzed by non-reducing 10% SDS-PAGE and stained with Coomassie brilliant blue. Tf, transferrin. (C) The same gel as in (B) transferred to nitrocellulose and probed with antibodies to transferrin (Tf), Hpr, ApoL-1, and ApoA-1.

Previous analysis of the protein composition of TLF, purified by the two-step procedure, demonstrated the presence of ApoA-1, ApoL-1, Hpr dimer, and Hpr tetramer (Figure 1A) [14]. Comparison of proteins purified by the two procedures by SDS-PAGE and western blot analysis showed that the protein compositions were similar in both cases (Figure 1). This shows that high-salt density ultracentrifugation used in the two-step protocol does not dramatically change the protein composition of the TLF particle. Two differences were noticed. First, the ratio of Hpr dimer to tetramer was higher in samples purified using the single-step immunoabsorption method. Second, there was a reduction in sub-stoichiometric proteins in preparations of TLF purified by the two-step purification method. Specifically, a significant amount of Hp, albumin, and transferrin was detected by SDS-PAGE, western blotting, and liquid chromatography/mass spectrometry/mass spectrometry (LC-MS/MS) in the one-step purified material (Figure 1A–1C, Table 1). In addition to these serum proteins, other human serum proteins, including angiotensinogen and intellectin, were detected at low abundance by LC-MS/MS analysis (unpublished data). Notably, no Hb was detected in any of the TLF preparations.

**Table 1:**
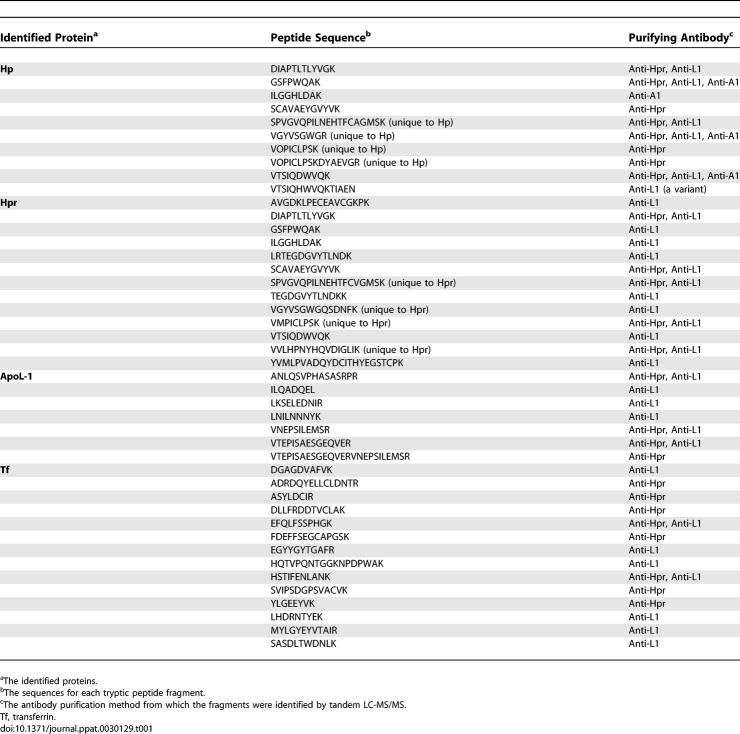
Mass Spectroscopy Analysis of TLF

In order to determine whether the protein composition of HDLs containing Hpr or ApoL-1 differed significantly, samples were purified from human plasma by the one-step immunoaffinity method using antibodies to Hpr, ApoL-1, or ApoA-1. All samples were analyzed by Coomassie staining, western blot, and LC-MS/MS (Figure 1; Table 1). The protein composition of samples purified by anti-Hpr or anti-ApoL-1 absorption was indistinguishable. The similar ratio of Hpr and ApoL-1, in the samples purified with anti-Hpr and anti-ApoL-1, indicates that most serum Hpr and ApoL-1 are assembled into the same HDL. The major proteins present in samples purified by anti-ApoA-1 immunoaffinity chromatography were ApoA-1, Hp, and transferrin. Only trace amounts of ApoL-1 and Hpr were detected in these samples, consistent with TLF being a minor subclass of human HDL [6]. These findings rule out the possibility that a significant amount of human plasma Hpr or ApoL-1 is free in the circulation and suggests a pathway for co-assembly of these two apolipoproteins into the same HDL particle.

### Analysis of Differentially Purified TLF Lytic Activity

**Figure 2:**
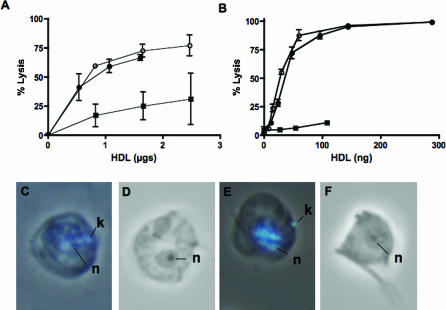
Lysis and Morphology of *T. b. brucei* Killed with Purified HDL (A) Human HDLs were purified by a single-step immunoaffinity method with either anti-Hpr, anti-ApoL-1, or anti-ApoA-1. The specific activity for *T. b. brucei* killing was similar for TLF purified with the single-step protocol with either anti-Hpr (closed circles) or anti-ApoL-1 (open circles) (∼0.5 μg/ml killed 40% of the trypanosomes in our standard assay). The specific activity of samples purified with single-step immunoabsorption with anti-ApoA-1 (closed squares) was over 10-fold reduced in specific activity for *T. b. brucei* killing. (B) TLF purified by a two-step method using both high-salt density gradient ultracentrifugation and immunoaffinity absorption. TLF first subjected to high-salt gradient ultracentrifugation was 10-fold more toxic to *T. b. brucei* than TLF purified by the single-step immunoabsorption directly from serum. Similar specific activities were obtained when the second immunoaffinity step was conducted with either anti-Hpr (closed circles) or anti-ApoL-1 (open circles). The specific activity of HDLs purified with anti-ApoA-1 (closed squares) was over 100-fold lower. (C–F) Cell morphology of *T. b. brucei* treated with TLF purified by single-step immunoaffinity with anti-ApoL-1 or anti-Hpr. Following incubation at 37 °C for 2 h, cells were either smeared on microscope slides and methanol fixed (C and E) or directly viewed following imbedding in 1% agarose (D and F). Incubation with TLF purified either by anti-Hpr (C and D) or anti-ApoL-1 (E and F) immunoabsorption showed similar morphologies. Prior to lysis all cells progressed through a series of morphological changes that included transition to a kite-shaped form and continued swelling until lysis. The position of the kinetoplast (k) and nucleus (n) was determined by DAPI staining in the fixed preparations (C and E). The nucleus (n) was clearly visible in the unfixed cells (D and F). doi:110.1371/journal.ppat.0030129.g002

The specific activity of TLF purified by the one-step method using antibodies to either Hpr or ApoL-1 was similar. In our standard lysis assay, 0.5 μg resulted in 40% lysis in 2 h at 37 °C (Figure 2A). Human plasma samples purified with anti-ApoA-1 were also trypanolytic, but the specific activity for killing was reduced over 10-fold (Figure 2A). The low specific activity of the ApoA-1-purified samples was due to the relatively low ratio of TLF to non-lytic HDL in this preparation. This is consistent with previous estimates of TLF abundance, suggesting it represents less than 0.1% of total human serum HDL [6,14].

When plasma samples were purified by the two-step protocol that included high-salt density ultracentrifugation followed by either anti-ApoL-1 or anti-Hpr immunoaffinity purification, the specific activity for trypanosome killing was approximately 10-fold higher than samples purified by the one-step methods (0.025 μg and 0.04 μg giving ∼50% lysis, respectively) (Figure 2B). The increased lytic activity of the samples purified by the two-step procedure may be due to the increased purity of these samples. This is because gradient centrifugation employed in the two-step purification process reduces the amount of contaminating, high-abundance serum proteins, such as albumin and transferrin (Figure 1A). In addition, Hp, a potent inhibitor of TLF killing of *T. b. brucei* [9], is weakly associated with all human HDL and is effectively removed by high-salt density ultracentrifugation (Figure 1A). The mechanism of Hp inhibition of human serum lysis of *T. b. brucei* is not known; based on results presented later, inhibition may be a direct consequence of Hp binding to free Hb in serum.

Two different morphological phenotypes have been reported for human HDL killing. One is defined by lysosomal membrane breakdown, cell swelling, and trypanosome death [13,24,30,35,36]. The other, recently reported by Pays and co-workers, involves extensive lysosome swelling, creating a large cytosolic vacuole that is proposed to exert enough pressure on the plasma membrane that the cell ruptures [8,10,25]. Plasma samples purified by either anti-Hpr or anti-ApoL-1 immunoaffinity produced identical morphological changes in *T. b. brucei* preceding lysis (Figure 2C–2F). After a lag phase of approximately 20 min, the overall morphology of the cells changed. Swelling was accompanied by an overall change in the shape of the cells, first resulting in a kite-like appearance followed by continued swelling and rounding of the cells until the cells ruptured. The formation of a large cytoplasmic vacuole was not observed. In live cell imaging, the nucleus, nucleolus, flagella, and other organelles are visible (Figure 2D and 2F). The morphology of *T. b. brucei* treated with intact human plasma produces the same morphological changes, leading to *T. b. brucei* lysis, like those seen with purified TLF (Figure 8C). The reason for the discrepancies between the morphological changes observed by the Pays lab and those observed by us and others following treatment with either purified TLF or intact human serum/plasma remains unresolved.

### TLF Binding to Hb

**Figure 3:**
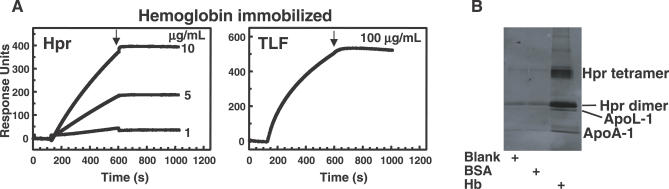
Binding of Purified Hpr and TLF to Hb (A) SPR analysis of the binding of purified Hpr (left panel) and TLF purified by the two-step procedure (right panel) to immobilized Hb A_0_. The concentration of Hpr and TLF is shown to the right of the binding curves. The BIAevaluation 4.1 software estimated a K_d_ of 2–5 nM for the Hpr–Hb complex. Arrows indicate the beginning of the dissociation curves. (B) Silver-stained SDS-polyacrylamide (8%–16% gradient) gel of proteins eluted from Hb-coupled Sepharose (Hb), BSA-coupled Sepharose (BSA), or underivatized Sepharose (blank) after incubation with purified TLF. doi:110.1371/journal.ppat.0030129.g003

Previous studies suggested Hpr, despite being highly homologous to Hp, did not bind Hb [12,30]. It is likely that Hpr–Hb complexes were not detected in these studies because of the presence of mild, non-ionic detergents in the immunoprecipitation assays. More recently, it has been shown that native and recombinant Hp and recombinant Hpr bind Hb with high affinity and that Hb-coupled Sepharose can precipitate Hpr-containing HDLs from plasma [31]. Although our analysis of TLF revealed the presence of Hpr, no Hb was detected in any of the purified HDL preparations. In order to determine whether native TLF and purified native Hpr could bind Hb, surface plasmon resonance (SPR) analysis was performed with purified preparations of native TLF, Hpr, and Hb. TLF and Hpr both bound immobilized Hb with high affinity (the K_d_ for the Hpr–Hb interaction was 2–5 nM as estimated by SPR) (Figure 3A). The horizontal progress of the curves representing the dissociation phase (after arrows in Figure 3A) indicates an almost irreversible binding of Hpr as well as binding of TLF to Hb.

To ensure that the SPR response observed upon incubation with TLF (Figure 3A, right panel) could not be attributed to binding between Hb and Hpr released from the TLF particle, binding of purified TLF to Hb was also studied in a pull-down assay. As revealed in Figure 3B, Hb-coupled Sepharose beads specifically bound the entire TLF particle containing Hpr as well as ApoL-1 and ApoA-1. Consistent with the recent finding that recombinant Hpr in complex with Hb does not bind the human Hp–Hb receptor CD163, the complex between native Hpr and Hb did not bind to immobilized purified human CD163 in the SPR analysis (unpublished data) [31,33].

### Hb Involvement in TLF Killing of *T. b. brucei*

**Figure 4:**
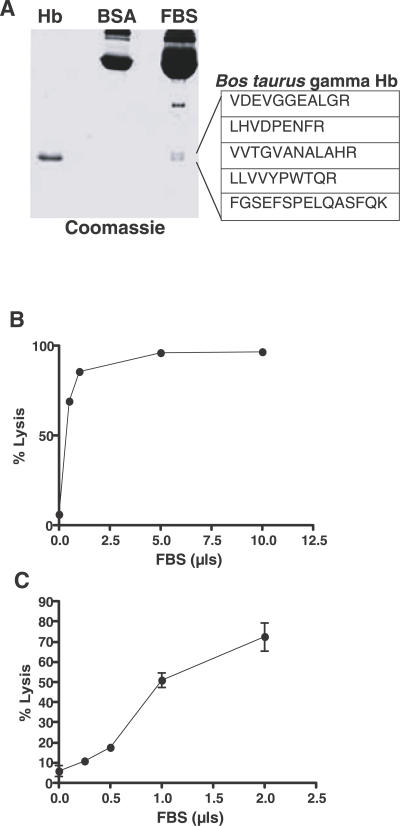
Effect of FBS on TLF Killing of *T.b. brucei* (A) FBS (1 μl), BSA (1 μg), and Hb (1.0 μg) were fractionated on 15% SDS-PAGE gels and stained with Coomassie. The presence of Hb in FBS was verified by mass spectroscopy of the appropriate sized bands. Both Bos aurus alpha and gamma subunits of Hb were identified, and five of the gamma globin tryptic fragments are shown. (B) FBS is required for maximum in vitro lysis of *T. b. brucei* with highly purified TLF. FBS was titrated into a 300-μl assay containing 1% BSA. Addition of 5 μl FBS restored lytic levels to those seen in standard lysis assays [8]. (C) The effect of FBS addition is concentration dependent. TLF used in these studies was purified by the two-step procedure.

The observation that Hb binds to purified Hpr and TLF led us to re-investigate the role of Hb in TLF-mediated killing of *T. b. brucei*. Since fetal bovine serum (FBS), a component of our standard trypanosome assay, typically contains small amounts of Hb released by hemolysis during serum preparation, we modified the standard trypanosome lysis assay to eliminate FBS (Figure 4A). The killing activity of TLF was drastically reduced when bovine serum albumin (BSA) was substituted for FBS in our in vitro lysis assay. Addition of FBS restored maximal tyrypanosome killing activity in the assay (Figure 4B and 4C).

**Figure 5:**
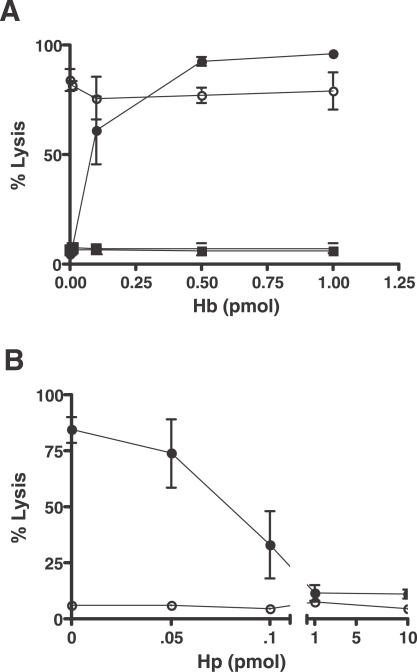
Hb Is the Co-Factor in FBS Necessary for Maximal TLF Killing of *T. b. brucei* (A) Titration of Hb into in vitro lysis reactions containing highly purified TLF. Samples contained TLF (1.5 units) in 10% FBS (open circles); TLF (1.5 units) in 1% BSA and 1% glucose (closed circles); Hp in 1% BSA and 1% glucose without TLF (closed squares); and no TLF (Hb only) (open squares). In the presence of TLF, Hb addition results in a concentration-dependent increase in *T. b. brucei* killing. No increase is seen when Hb is added to FBS-containing reactions because FBS contains approximately 0.5 μg/μl of Hb. (B) Addition of Hp inhibits Hb-dependent killing by TLF. Hp was titrated into reactions containing TLF (1.5 units), 1% BSA and 1% glucose, and 0.1 pmol of Hb (closed circles). Addition of Hp and Hb (0.1 pmol) to *T. b. brucei* in the absence of TLF had no effect on cell viability (open circles). TLF used in these studies was purified by the two-step procedure.

To determine whether Hb was the co-factor supplied by the FBS in the in vitro lysis reactions, we titrated Hb into the modified serum-free assay (Figure 5A). Hb restores the killing activity of TLF, while the addition of Hb alone is non-toxic to *T. b. brucei* even at very high concentrations (Figure 5A; Figure S1). Furthermore, if the Hb-binding protein, Hp, is added to the reactions, it inhibits lysis in a concentration-dependent fashion (Figure 5B). Together, these results indicate that Hb is a necessary co-factor for maximal TLF killing of *T. b. brucei* and that direct binding of Hb to Hpr may be required.

### TLF–Hb Binding to Trypanosomes

**Figure 6:**
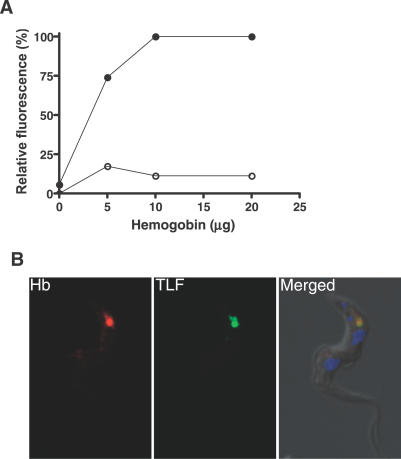
Hb Is Required for Binding and Uptake of TLF (A) Alexa Fluor 488–labeled TLF was incubated with trypanosomes and increasing amounts of Hb. Binding was measured by flow cytometry and the relative mean fluorescence intensity determined following binding for 1 h at 4 °C. In the absence of Hp, there is a dose-dependent increase of binding of Alexa Fluor 488–labeled TLF in the presence of Hb (closed circles). Addition of Hp inhibited TLF–Hb binding to *T. b. brucei* (open circles). (B) Uptake of TLF–Hb by *T. b. brucei*. Trypanosomes were pretreated with chloroquine for 30 min to block acidification of the lysosome and activation of TLF, followed by the addition of Alexa Fluor 594–labeled Hb and Alexa Fluor 488–labeled TLF. Cells were incubated for an additional hour at 37 °C. The TLF–Hb complex was endocytosed and traffics to the lysosome (Figure S2B). TLF used in these studies was purified by the two-step procedure.

Hb could stimulate trypanosome killing either a) by increasing the affinity of TLF for the trypanosome receptor, b) by playing a direct role in the lytic process, or c) by a combination of the above. To test whether Hb was necessary for trypanosome binding, Alexa Fluor 488-labeled TLF was incubated with trypanosomes at 4 °C for 1 h, cells were washed, and then cell-associated fluorescence was measured by flow cytometery (Figure 6A). Addition of Hb increased binding of TLF to *T. b. brucei.* Maximum stimulation of binding was reached at a concentration of approximately 30 μg/ml, which represents a 1:10 molar ratio of TLF to Hb, assuming an average molecular mass of 500 kDa for TLF [6]. Addition of an equimolar concentration of Hp to Hb severely inhibited binding of TLF to cells, resulting in binding levels similar to the TLF binding observed in the absence of Hb, either by sequestering all available Hb or by competition for the trypanosome TLF–Hb receptor. These experiments suggest that Hb binding to TLF stimulates receptor-mediated binding to *T. b. brucei*, and that TLF–Hb binding to the receptor is specific and saturable.

Killing of *T. b. brucei* by TLF requires trafficking of the toxin to the acidic trypanosome lysosome [22,24]. To determine whether both TLF and Hb co-localize to the lysosome, Alexa Fluor 488–conjugated TLF and Alexa Fluor 594–conjugated Hb were incubated with trypanosomes. When TLF or Hb alone was incubated with cells, no intracellular fluorescence was observed (Figure S2A). Co-incubation of TLF and Hb resulted in the rapid uptake and intracellular co-localization to the lysosome (Figures 6B and S2B). These results illustrate that TLF needs to be associated with Hb in order to be endocytosed by *T.b. brucei*, and that both TLF and Hb traffic to the lysosome of the cell.

### TLF–Hb Toxicity to Trypanosomes

**Figure 7:**
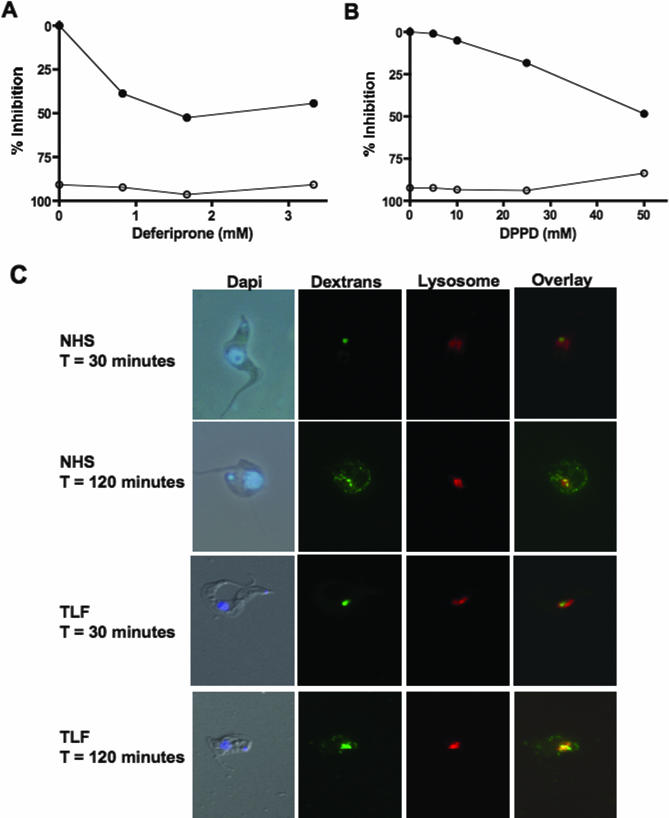
TLF–Hb Induces Iron-Dependent Lysosomal Membrane Breakdown (A) *T. b. brucei* was treated with increasing concentration of the iron chelator deferiprone in the presence (closed circles) and absence (open circles) of TLF–Hb. The inhibition of trypanosome lysis by deferiprone is indicated. We normalized the data to indicate that the addition of no deferiprone gave zero inhibition of TLF–Hb lysis after 2 h at 37 °C. (B) Trypanosomes were treated with increasing concentrations of the antioxidant DPPD in the presence (closed circles) or absence (open circles) of TLF–Hb. The inhibition of trypanosome lysis by DPPD is indicated. We normalized the data to indicate that the addition of no DPPD gave zero inhibition of TLF–Hb lysis after 2 h at 37 °C. (C) Effect of human serum and TLF–Hb treatment on *T. b. brucei* lysosomal membrane structure. In order to label the lysosome with a size-selective membrane-permeable marker, *T. b. brucei* was pre-incubated for 30 min at 37 °C with fluorescein-conjugated dextrans (500 kDa). Normal human serum (NHS) or purified TLF–Hb was then added. After 30 or 120 min, cells were formaldehyde fixed and stained with anti-p67 to localize the lysosomal membrane. After treatment with normal human serum or TLF–Hb for 30 min, dextrans are localized to a single cytoplasmic structure that stained with an antibody against the lysosomal marker p67. Trypanosomes treated for 120 min with either normal human serum or TLF–Hb continued to show p67 staining largely restricted to a single structure in the cell, but the 500-kDa dextrans are widely distributed in the cells, indicating a breakdown in lysosomal membranes. TLF used in these studies was purified by the two-step procedure.

Heme is toxic to trypanosomes presumably through production of free radicals that lead to lipid peroxidation of trypanosome membranes [37]. Previous studies suggested that the toxicity of TLF might involve lysosomal membrane peroxidation [15,24,30]. Since Hb traffics with TLF to the trypanosome lysosome, we asked whether Hb could initiate a Fenton-like reaction. Fenton chemistry requires ferrous iron, hydrogen peroxide, and low pH to produce hydroxyl radicals that peroxidate lipid and can lead to membrane degradation and cell death [38]. All of these conditions are present if TLF transports Hb to the trypanosome lysosome. To test whether TLF–Hb induces a Fenton reaction, we performed lysis assays in the presence of the iron chelator deferiprone and in the presence of the free radical scavenger N,N′-diphenyl-1,4-Benzenediamine (DPPD) (Figure 7A and 7B). Both iron chelation and the scavenging of free radicals inhibited lysis by about 50%.

To further investigate the mechanism of TLF killing, we returned to morphological analysis of TLF–Hb-treated trypanosomes. We have shown that TLF killing of *T. b. brucei* results in cell swelling, changes in overall cell morphology, and eventually lysis (Figure 2C–2F) [24]. Consistent with earlier electron microscopy of cells treated with gold-conjugated TLF [24,36], these results suggest that TLF–Hb causes the breakdown of lysosomal membranes. To determine whether TLF–Hb caused extensive lysosomal membrane breakdown, *T. b. brucei* was incubated with fluorescein-labeled 500-kDa dextrans, which trafficked to the lysosome by bulk phase endocytosis (Figure 7C). After the cells were pre-loaded with dextrans for 30 min, TLF–Hb or human plasma was added to the cells. After 30 min of exposure, the fluorescent dextrans were found predominately in a single vesicle co-localizing with the lysosomal membrane marker p67. By 120 min post-treatment with either TLF–Hb or human serum, the dextrans were visible throughout the cell, while the p67 remained predominantly associated with the singular lysosome. Since the fluorescently labeled dextrans escape the lysosome in the presence of TLF, the lysosomal membrane must be open to allow release of the large dextrans. The increased intensity of the fluorescein-labeled dextrans at 120 min post TLF treatment is likely due to their release into the neutral pH of the cytoplasm [39]. Incubation with normal human plasma also resulted in release of pre-loaded dextrans into the cytoplasm prior to trypanosome lysis (Figure 7C). These results show that TLF–Hb causes lysosomal membrane breakdown prior to cell lysis, as does normal human serum.

## Discussion

We have identified Hb as a critical co-factor in the killing of *T. b. brucei* by human TLF. Analysis of purified TLF indicates that TLF is a subclass of human HDL containing ApoA-1, and two primate-specific apolipoproteins, Hpr and ApoL-1. Hpr binds human Hb with high affinity, and the formation of the TLF–Hb complex enhances binding to the trypanosome TLF receptor and triggers lysosome breakdown by an iron-dependent, free radical–mediated pathway. Despite the role of Hb in TLF binding and toxicity, no Hb is detected in TLF prepared from healthy human donors. This indicates that activation of TLF occurs upon the release of free Hb in the circulation during intravascular hemolysis.

### TLF Contains Both Hpr and ApoL-1

A major question concerning the killing of *T. b. brucei* by human serum has centered around whether the toxin was Hpr, ApoL-1, or both of these proteins [10,14,25,40]. In this paper, the protein composition of human TLF was determined by western blot and LC-MS/MS. While several studies have previously identified protein components of this subclass of trypanolytic human HDL, all have used samples that have been subjected to lengthy purification protocols involving high-salt density ultracentrifugation [6,34]. We were concerned that these procedures would dissociate proteins from TLF that might provide information on the native composition of this innate killing factor and on the mechanism of killing.

When TLF was isolated directly from freshly prepared human plasma by immunoaffinity chromatography with antibodies against either human ApoL-1 or Hpr, the major protein components were identical. The specific activity and morphology of *T. b. brucei* lysis was also identical. This analysis indicates that Hpr and ApoL-1 are assembled into the same HDL particle and that the amount of Hpr or ApoL-1 found free in the human circulatory system is extremely low. Thus, the native toxin in humans is not ApoL-1 or Hpr alone but is an HDL particle containing both of these apoliproteins. This is consistent with previous studies showing that Hpr and ApoL-1 were present in the same HDL and both were required for maximal trypanosome killing [14]. In addition, recent analysis of the trypanolytic activity of human serum from individuals deficient in either ApoL-1 or Hpr suggests that both proteins play distinct and important roles to achieve maximal lytic activity [40].

### The Role of Hb in *T. b. brucei* Killing by TLF

Several early studies reported that TLF did not bind Hb [12,30], but recently it was shown that recombinant Hpr bound Hb with high affinity [31]. Consistent with these results, we found that purified native TLF and purified native Hpr bound Hb with high affinity (Figure 3). Nevertheless, MS analysis failed to detect Hb as a component of purified TLF, most likely because the low levels of Hb that are released into the plasma of individuals with normal erythrocyte turnover are instantly bound by Hp, which is present in large excess compared to Hpr (the concentration of Hp is roughly 10- to 100-fold higher than Hpr in normal human serum) [12,31]. The rapidly formed Hp–Hb complex is then removed by CD163. Hence, despite its high affinity for Hb, Hpr may only compete well for free Hb under physiological conditions where Hp levels were low. As we discuss below, infection by trypanosomes causes declines in Hp levels in animals [41–44].

In order to evaluate the role of Hb as a co-factor in TLF-mediated killing of *T. b. brucei,* we developed an in vitro lysis assay without FBS, a standard component of our in vitro trypanosome lysis assays, and a source of contaminating free Hb (Figure 4A). We found that substitution of BSA for FBS in lysis assays dramatically reduced the activity of TLF, but that maximal activity could be restored by addition of human Hb to the assays (Figure 5A).

Hb stimulation of *T. b. brucei* killing by TLF is due to both enhanced binding to the trypanosome cell surface receptor for TLF and direct cytotoxicity of lysosomally localized Hb (Figures 6 and S2B). The basis for the enhanced trypanosome receptor recognition of the TLF–Hb complex is unknown, but the mammalian CD163 receptor shows a similar binding preference for the Hp–Hb complex [32,33]. Our studies show that not only is trypanosome binding of TLF stimulated by Hb association but that the trafficking of both TLF and Hb to the lysosome is enhanced (Figures 6 and S2B).

The mechanism of TLF-induced cell lysis is controversial, but studies from our lab have shown that both freshly prepared normal human serum and TLF produce peroxidated lipids in *T. b. brucei,* suggesting a mechanism of killing [30]. Based on the findings reported here, we propose that Hb, bound to Hpr within the TLF particle, contributes directly to the established ApoL-1-mediated toxicity of TLF [8,10,28,29,40]. Following binding and lysosomal localization, Hb may contribute iron that reacts with H_2_O_2_ in a Fenton-based reaction that leads to free radical formation and lysosomal membrane breakdown (Figure 7) [9,15,24,45]. Interestingly, earlier studies using an in vitro TLF assay containing 0.2% BSA, and no Hb, did not kill *T. b. brucei* by a Fenton-based mechanism ([46]).

The morphological analysis of *T. b. brucei* treated with TLF–Hb showed cells swelling into a kite-shape prior to the bursting. We were able to directly test the permeability of the lysosomal membrane following TLF–Hb treatment by pre-loading cells with defined size (500 kDa) fluorescein-conjugated dextrans then adding TLF–Hb (Figure 7C). Prior to lysis, dextrans were seen throughout the cytoplasm indicating that TLF–Hb caused the lysosomal membrane to breakdown, releasing the large fluorescein-conjugated dextrans. Based on the pharmacological inhibition of TLF–Hb killing by deferiprone and DPPD, we postulate that membrane lipid peroxidation plays a major role in lysosomal membrane breakdown followed by cell lysis. This does not argue against a role of ApoL-1 in TLF killing of *T. b. brucei* [8,10,19,25–28]. Recombinant and native ApoL-1 are toxic to *T. b. brucei* and the evidence that it creates ion pores in trypanosome membranes is convincing [10].

### Activation of the Innate TLF Response by Hb

Does Hb stimulation of TLF binding and killing of *T. b. brucei* occur in humans, and is this important in protection against these parasites? In vitro studies reported here leave little doubt that Hb association with Hpr enhances TLF binding to and killing of *T. b. brucei*. However, when TLF is isolated from normal human donors, Hb is absent. This apparent paradox can be explained when one carefully considers the early physiological events associated with trypanosome infection. During infection of animals by African trypanosomes, there is substantial hemolysis, and in some cases, the hematocrit can drop by as much as 50% [42–44]. The exact cause of hemolysis is not known, but blood cell production increases during infection, so the decrease in hematocrit is not due to decreased erythrocyte production but rather to hemolysis [47]. Hemolysis results in the release of large quantities of Hb, most of which will be bound and removed by circulating Hp [42,48]. During a trypanosome-induced acute phase response in calves, levels of Hp are reduced to undetectable levels 8 d post-infection, presumably due to removal of Hp from the circulation after Hp–Hb complexes have formed [42]. In mice, Hp levels initially increase after trypanosome infection, and then decline [48,49].

Consistent with the studies in animals, human trypanosome infections also result in substantial hemolysis and release of free Hb [50]. While Hp initially binds all free Hb, the clearance of the Hp–Hb complex would result in a significant drop in Hp levels to the point where Hpr would become a major Hb-binding protein. Thus, as Hp levels decrease and TLF–Hb levels increase, there may be a substantial stimulation of TLF activity.

A second factor may influence the formation of TLF–Hb complexes in the circulation of humans. Previous studies have reported the incidence of both genetic and phenotypic ahaptoglobinemia in African populations, perhaps due to reduced severity of malaria infection in Hp-negative individuals [51–53]. In some regions where malarial infections are prevalent, the frequency of ahaptoglobinemic individuals can be as high as 48% [51,54,55]. In these individuals, Hpr-associated TLF could be the primary Hb-binding protein.

We have previously postulated that ApoL-1 and Hpr act synergistically in *T. b. brucei* killing [14]. We now propose an avenue by which TLF activity is increased when Hpr and ApoL-1 are present in the same HDL particle. First, Hb binding to TLF stimulates endocytosis of TLF–Hb by increased receptor affinity. Second, TLF–Hb provides iron that initiates Fenton chemistry, leading to lysosomal membrane breakdown while ApoL-1 forms pores in membranes, disrupting the ability of the cell to regulate osmosis.

The results presented here support the hypothesis that formation of the Hpr–Hb complex within the TLF particle plays a role in human serum killing of *T. b. brucei*. Hb seems to induce increased binding of TLF to the parasite and contributes directly to toxicity in the lysosome. This suggests that parasite-induced hemolysis stimulates innate immunity against the parasite in humans.

## Materials and Methods

### Human serum preparation and immunoaffinity chromatography.

Blood was collected from healthy human volunteers and was maintained on ice or at 4 °C throughout the fractionation procedure. Plasma was separated from blood cells by centrifugation at 3,500 rpm in a SLA-3000 rotor (Sorvall, http://www.thermo.com/) for 10 min at 4 °C. The plasma supernate was removed and re-centrifuged in an SS-34 rotor (Sorvall) at 9,500 rpm for 10 min at 4 °C to remove any residual blood cells. Plasma was kept on ice for no more than 3 h prior to antibody affinity chromatography.

Monoclonal antibodies were raised against TLF particles, and the specificity of the antibodies for Hpr and ApoL-1 has been previously described [14]. The monoclonal antibodies were purified from mouse ascites by Protein G affinity chromatography according to the manufacturer's recommendations (Pierce Biotechnology, http://www.piercenet.com/). Affinity-purified polyclonal antibodies against human ApoA-1 were purchased from Rockland Immunochemicals (http://www.rockland-inc.com/). The three antibodies (200 μg) were coupled to Affigel 15 (50 μl) (Bio-Rad, http://www.bio-rad.com/) according to the manufacturer's recommendations and 100 μl of the AffiGel slurry (corresponding to approximately 200 μg of antibody) was transferred to 1.5-ml microfuge tubes. Human plasma (1 ml) was added to each antibody containing tube and incubated for 30 min at 4 °C on a rotating platform. AffiGel/antibody resin was recovered by centrifugation for 90 s at 1,000*g* at 4 °C and the immuno-depleted plasma discarded. Additional 1-ml samples of plasma were added to each sample and immuno-depletion steps were repeated until 5 ml of plasma had been treated. The AffiGel/antibody resin was then washed seven times with 1 ml of PBSE (137 mM NaCl, 2.7 mM KCl, 10 mM Na_2_HPO_4_, 10 mM KH_2_PO_4_, 3 mM EDTA). Following the washes, the AffiGel/antibody resin was treated with 200 μl of 100mM glycine (pH 2.0) for 5 min at 4 °C to elute bound human plasma proteins. The eluate was dialyzed against PBSE at 4 °C prior to analysis for protein composition and trypanolytic activity.

### Western blot analysis of TLF.

Following elution from anti-ApoL-1, anti-Hpr, and anti-ApoA-1 AffiGel resin, samples were precipitated with 100% ice-cold acetone and proteins were separated on a 10% SDS-polyacrylamide gel under non-denaturing conditions. Gels were either Coomassie stained or western blotted onto nitrocellulose. For western blot analysis, membranes were blocked with 5% milk in TTBS (20mM Tris, 500mM NaCl, 0.05% Tween-20) and were probed with antibodies diluted with 5% milk in TTBS: Hpr (1:10,000), ApoL-1 (1:10,000), ApoA-1 (1:5000), Hb (1:2000), and transferrin-Hrp (1:2000). After incubation with secondary antibodies (goat anti-rabbit and goat anti-mouse at 1:10,000 in all cases except transferrin), (Pierce Biotechnology) proteins were detected following reaction with ECL Plus (GE Healthcare, http://www.gelifesciences.com/) and were visualized by autoradiography. Protein concentrations were determined by Bradford assay (Bio-Rad).

### LC-MS/MS.

For mass spectroscopy analysis, protein bands were excised from Coomassie-stained gels, trypsin digested, and fractionated by reverse phase chromatography (C-18 PepMap100, 75 um ID × 15 mm, 3-um particle size, LC Packings/Dionex, http://www.dionex.com/). The column eluate was introduced onto a QSTAR XL mass spectrometer (Applied Biosystems, http://www.appliedbiosystems.com/, and MDS Sciex, http://www.mdssciex.com/) by electrospray ionization. Ions were selected and fragmented using a standard information dependent acquisition method. An ion had to be assigned a charge in the +2 to +4 range to be considered a candidate for fragmentation. Identification of proteins was performed using ProID software (Applied Biosystems) and experimental spectra were matched against *in silica* trypsinizations of the NCBI non-redundant database.

### *T. b. brucei* cultivation and lysis assays.

Bloodstream *T. b. brucei* (Lister strain 427, MiTat 1.2) were cultured in HMI-9 media supplemented with 10% FBS (heat inactivated, 58 °C for 30 min). Trypanolytic activity of fractionated HDLs was determined by in vitro microscopic assays as previously described [6]. All samples were dialyzed exhaustively against PBSE, at 4 °C, prior to incubation with the parasites (1 × 10^7^ cells per 300-μl assay) for 2 h at 37 °C in HMI-9 media containing 10% FBS. To determine whether FBS contributed to TLF-mediated killing of *T. b. brucei* some lysis assays contained 1% BSA and 1% glucose without intact FBS. Hb A_0_ and Hp 1–1 were obtained from Sigma-Aldrich (http://www.sigmaaldrich.com/). In this paper and in previous papers, we define a lytic unit of activity as the amount of material necessary to lyse 50% of the parasites, in a 300-μl assay containing 1 × 10^7^ cells after incubation for 2 h at 37 °C [8]. The iron chelator 1,2-dimethyl-3-hydroxypyrid-4-one (deferriprone) was used in the chelation inhibition studies and DPPD was the antioxidant used.

### Cell imaging.

Lysis of live parasites was visualized by the addition of TLF to *T. b. brucei* imbedded in 1% low melting point agarose (Sigma) made with PBSE containing 1% glucose following 2 h of incubation at 37 °C. Samples were prepared for microscopy by the addition of 10 μl of low melting point agarose to 10 μls of *T. b. brucei* at a final concentration of 1 × 10^7^/ml. Both the agarose and trypanosome samples were maintained at 37 °C, gently mixed, and transferred to a microscope slide. To further reduce the motility of trypanosomes, the slide was chilled at 4 °C for 10 min and then imaged using a motorized Zeiss Axioplan2 and an MRm camera interfaced with the Axiovisions 4.4 software (Zeiss, http://www.zeiss.com/). Methanol-fixed samples were also visualized. Cells incubated with TLF samples as above were washed with PBSE containing 10% FBS, then resuspended and smeared on a microscope slide. Slides were rapidly air dried, and cells were fixed for 5 min in methanol (−20°C). Following methanol fixation, slides were air dried and mounted with 4',6-diamidino-2-phenylindole (DAPI) containing antifade reagent ProlongGold (Invitrogen, http://www.invitrogen.com/) and imaged as above.

### Lysosomal morphology studies.

Trypanosomes were resuspended at a concentration of 1 × 10^7^/ml in F12-FBS and were incubated with 300 μg/ml 500-kDa fluorescein-labeled dextrans (Invitrogen) for 30 min at 37° C. Cells were then washed three times with 1X PBSE and were incubated at 3 × 10^6^/ml in F12-FBS with either 2 units of normal human serum (1.7% total human serum) or 2 units of purified TLF (0.017% TLF), and cells were examined after 30 min or 2 h. Aliquots were treated with 0.001% formaldehyde on ice for 5 min, rinsed with PBS, and resuspended in PBS-10% FBS. Cells were then smeared onto a slide and air dried. For co-localization with anti-p67, cells were rehydrated in PBS-10% FBS and incubated in 1:1000 anti-p67 (a gift of Jay Bangs, University of Wisconsin, Madison,Wisconsin, United States) for 1 h at room temperature, followed by staining with a goat anti-mouse secondary antibody labeled with Alexa Fluor 594 (Invitrogen). Slides were rinsed in PBS containing 1% glucose, air dried, and viewed using a motorized Zeiss Axioplan2 and an MRm camera interfaced with the Axiovisions 4.4 software (Zeiss).

### Hb binding.

SPR analysis was conducted essentially as described in [31] except that native TLF and Hpr were used in the binding assays instead of recombinant Hpr. Hpr and TLF were purified from fresh human plasma using the two-step purification method [14]. In the case of Hpr purification, human HDLs were solubilized using 10mM CHAPS and purified using an anti-Hpr column as described [14]. The K_d_ for the Hpr–Hb interaction was estimated by the BIAevaluation 4.1 software (http://www.biacore.com/lifesciences/index.html) using a Langmuir 1:1 binding model.

For precipitation experiments, purified TLF was incubated with Hb-coupled Sepharose, BSA-coupled Sepharose, or underivatized Sepharose. After extensive washing in a solution containing 2 mM CaCl_2_, 1 mM MgCl_2_, 10 mM Hepes, and 140 mM NaCl (pH 7.8), bound proteins were eluted in SDS-containing sample buffer and visualized by SDS-PAGE.

### TLF binding and localization.

Binding studies of TLF to trypanosomes was conducted with Alexa Fluor 488 (Invitrogen) labeled TLF, and Hb and Hp noted above. Trypanosomes were incubated with labeled TLF, unlabeled Hb, and Hp at 4 °C for 1 h. The cells were then washed three times at 1,400*g* for 7 min at 4 °C. The cells were resuspended in 2% formaldehyde at 4 °C until measured by flow cytometry using the CyAn ADP (Dako, http://www.dako.com/) and analyzed using FlowJo software (TreeStar, http://www.treestar.com/). Immunofluoresence microscopy was employed to localize TLF and Hb in the cell. Alexa Fluor 488 (Invitrogen)–labeled TLF and Alexa Fluor 594 (Invitrogen)–labeled Hb were incubated with trypanosomes pre-incubated with 50 μM chloroquine for 30 min. TLF and Hb were added at the concentrations noted in Figure 7. LysoTracker Red DND99 (Invitrogen) was used to identify the lysosome. These studies were done on a Zeiss Axioplan and the images analyzed using IPLab Spectrum version 3.9.4r2 from Scanalytics–BD Biosciences (http://www.scanalytics.com/)

## Supporting Information

Figure S1 Hb Is Necessary for Maximal TLF Killing of *T. b. brucei*
Trypanosome killing by TLF in the presence (open circles) and absence (closed circles) of Hb. Hb alone is not toxic to *T. b. brucei* at the concentrations tested (open squares). TLF used in these studies was purified by the two-step procedure.
(25 KB PPT)

Figure S2 Neither Hb nor TLF Alone Are Efficiently Taken Up *by T. b. brucei*
(A) Alexa Fluor 594–labeled Hb or Alexa Fluor 488–labeled TLF was incubated with *T. b. brucei* for 1 h at 37 °C. No uptake of was seen at either 488 nm or 594 nm excitation.
(B) When cells were co-incubated with TLF and Hb, both TLF and Hb can be seen in the trypanosome lysosome by co-localization with LysoTracker. TLF used in these studies was purified by the two-step procedure.
(283 KB PPT)

## Abbreviations

ApoA-1: apolipoprotein A-I
ApoL-1: apolipoprotein L-1
BSA: bovine serum albumin
DPPD: N,N′-diphenyl-1,4-Benzenediamine
FBS: fetal bovine serum
Hb: hemoglobin
HDL: high-density lipoprotein
Hp: haptoglobin, Hpr, haptoglobin-related protein
LC-MS/MS: liquid chromatography/mass spectrometry/mass spectrometry
SPR: surface plasmon resonance
TLF: trypanosome lytic factor
